# Multilingualism is associated with small task-specific advantages in cognitive performance of older adults

**DOI:** 10.1038/s41598-023-43961-7

**Published:** 2023-10-07

**Authors:** Priscilla Achaa-Amankwaa, Ekaterina Münch, Hanna Miksch, Johanna Stumme, Stefan Heim, Mirjam Ebersbach

**Affiliations:** 1 Institute of Psychology, University of Kassel, Holländische Strasse 36-38, 34127 Kassel, Germany; 2 Institute of Anatomy I, Medical Faculty & University Hospital Düsseldorf, Heinrich Heine University Düsseldorf, Düsseldorf, Germany; 3 Institute of Neuroscience and Medicine (INM-1), Research Centre Jülich, Jülich, Germany; 4 Department of Psychiatry, Psychotherapy and Psychosomatics, Medical Faculty, RWTH Aachen University, Aachen, Germany

**Keywords:** Neuroscience, Psychology

## Abstract

The protective effects of multiple language knowledge on the maintenance of cognitive functions in older adults have been discussed controversially, among others, because of methodological inconsistencies between studies. In a sample of *N* = 528 German monolinguals and multilinguals (speaking two or more languages) older than 60 years, this study examined (1) whether speaking multiple languages is positively related to performance on tasks of interference suppression, working memory, concept shifting, and phonemic and semantic fluency, and (2) whether language proficiency and age of second language acquisition (AoA) are associated with cognitive performance of multilinguals. Controlling for education and daily activity, we found small cognitive benefits of speaking multiple languages on interference suppression, working memory, and phonemic fluency, but not on concept shifting and semantic fluency. Furthermore, no substantive correlations were found between language proficiency or AoA and cognitive performance. In conclusion, multilingualism appears to have small incremental effects on cognitive performance beyond education and daily activity in older age that are task-specific and widely independent of proficiency and AoA.

## Introduction

Aging is accompanied by the process of cognitive decline in functions such as (working) memory, aspects of inhibitory control^1,2^, visuo-spatial abilities, reasoning, information processing speed, and verbal fluency (for a review see, e.g. Salthouse^3^). Age-related neurocognitive changes such as gray matter depletion (i.e. brain atrophy, especially in frontal areas), and reduced neural network connectivity suggest neurodegenerative developments to account for this age-related decline^1,4,5^. However, this process is characterized by high interindividual variability and some people cope better with increased rates of brain atrophy while still maintaining high levels of cognitive functioning through age, whereas others become more susceptible to cognitive impairment. The theory of *cognitive reserve* explains the interindividual variability of age- or disease-related brain changes as a result of individual differences in how people have been drawing on cognitive processes throughout their lives or have been activating compensating processes^6^.

Considering the aging population, the detection of factors positively influencing cognitive maintenance in terms of contributing to the cognitive reserve across life has gained increasing importance. While factors such as intelligence, education, occupational complexity, and leisure and physical activity have been frequently identified as proxies of cognitive reserve in epidemiologic research^6^, the impact of bi- or multilingualism has been the subject of much controversy in recent years. Numerous studies have provided evidence for an advantage of speaking more than one language for a variety of cognitive systems targeting executive processes (see Bialystok^7^, for a review of studies across the lifespan) as well as evidence contesting such an effect^8–10^.

### The bilingual advantage hypothesis—current empirical evidence and controversy

The bilingual advantage hypothesis^11^ suggests that tasks requiring controlled processing are performed more efficiently by bilinguals than monolinguals and that bilingualism assists in compensating age-related losses in executive functions. This bilingual advantage is explained by a (lifelong) proficiency in managing and switching between two languages, calling for the continuous use of cognitive-executive resources that are essential for coordinating multiple languages^12,13^. Neuroimaging and experimental research have provided evidence that in bilinguals both languages are constantly active when listening to, reading, or planning speech in either language (for a review, see Kroll et al.^14^). As a consequence of the parallel activation of both languages, there is a competition for cognitive resources requiring bilinguals to regulate that competition to minimize cross-language intrusions in language use^14^. Unlike monolinguals, this means that bilinguals must (subconsciously) direct more attention to language representations and language processing^7^. More recently, a theory putting such attentional processes in the center of the bilingual advantage hypothesis has gained ground (for a detailed theoretical account, see Bialystok and colleagues^7,15^). According to this theory, the experiences linked to practicing multiple languages from an early age on lead to an adaptation of the attention system, which accommodates the demands posed by the need to navigate linguistic and nonlinguistic activities in bi- or multilingual environments.

The bilingual advantage hypothesis has chiefly been tested for executive functions, comprising higher-order cognitive abilities such as planning, decision-making, inhibition, and attention control^16–18^. Here, bilingual advantages have been observed consistently and most strongly in older adults, for whom the individual decline in cognitive performance becomes more evident. For instance, Bialystok et al.^19^ tested groups of young (in their twenties) and older (about 60–70 years) adult monolinguals and bilinguals on the Stroop task, measuring interference suppression, and a complex nonverbal working memory task. Both groups of bilinguals performed better than their monolingual peers, with the older group showing the most pronounced bilingual advantage. These findings are corroborated by a recent meta-analysis^20^, indicating that the effects of bilingualism on executive functions are often moderated by the participants’ age (i.e. more likely to be observed in participants older than 50 years). Importantly, this age effect is in line with research investigating the associations between bilingualism and cognitive reserve. The latter is thought to promote the decoupling of cognitive performance and brain structure that manifests in reduced effects of deteriorating brain structure on cognitive function^21^. Neuroprotective effects of bilingualism were demonstrated to enhance healthy aging processes and neuroplasticity^22–24^, and to reduce the risk of neuropathology such as dementia^25,26^.

The benefits of bilingualism have been investigated for various cognitive systems, linked to controlled information processing (for a review, see Bialystok^7^; for a Bayesian meta-analysis, see Grundy^27^). *Inhibitory control* is one of the key executive functions for which a positive effect of bilingualism has been found. Specifically, more successful conflict monitoring and interference suppression (i.e. the ability to ignore the effects of misleading information^15^) have been linked to bilinguals’ need to constantly restrict language production to the target language while suppressing the other in lexico-semantic processes^28^. These control processes generate a benefit for tasks demanding similar interference suppression, such as the Stroop task^19,29,30^.

Another area of executive control for which positive impacts of bilingualism have been reported is *working memory*. Meta-analytic evidence indicates a small to medium bilingual advantage in this domain^31,32^. The proposed rationale for the bilingual advantage in working memory^33^ is that greater working memory capacity is reflected in better attentional control whilst using the memory storage. This effect is explained through the central involvement of the working memory system in keeping momentarily relevant and contextually interfering information quickly retrievable. The latter also dovetails with the finding that increasing attentional demands of a task are associated with increasing differences in working memory performance between different language proficiency groups^34^.

Although less well researched than other areas, improved abilities of bilinguals have also been reported for *cognitive flexibility*, which includes the abilities (a) to switch flexibly and efficiently between tasks^35^, allowing to adapt behavior in response to changing environments, (b) to attend selectively to stimuli and information of interest^16^, and (c), more seldomly, to think abstractly and to acquire rules via inductive reasoning^36^. Connections between bilingualism and such skills are reflected, for instance, in bilinguals’ higher scores on the Card Sort Task and the Trail Making Test^20,37^.

Regarding *verbal abilities*, a fairly consistent pattern has emerged in which monolinguals perform better on tasks with increased verbal processing demands (concerning each of the bilinguals’ languages)^15^, whereas nonverbal tasks testing the same cognitive functions are performed better by bilinguals (see e.g. a comparison of bi- and monolinguals’ performance in spatial and verbal working memory tasks in a study by Luo and colleagues^38^). An exception to this has been the verbal (color-word) Stroop task, in which bilinguals perform better than monolinguals^19,30^. However, speaking multiple languages also comes at a cost for language production, as reflected in bilinguals’ prolonged reaction times and lower accuracy in lexico-semantic tasks^39,40^. These costs usually manifest in either comparable performances between mono- and bilinguals^41–43^ or in bilinguals’ lower performance in semantic (i.e. categorical) fluency tasks^10^. The disadvantages of bilingualism in these tasks might result from between-language interferences^44^. Furthermore, bilinguals’ lower performance in lexical access, such as in picture naming tasks, seems to be independent of language dominance, because it has been found for the first (or more proficient) and the second (or weaker) language^45^. In contrast, for phonemic (i.e. letter) fluency tasks, suggested to require higher executive demands than categorical fluency tasks, bilingual advantages have been reported^40,41^. For example, controlling for the vocabulary size of bi- and monolinguals, Luo et al.^43^ found no group difference in category fluency, but higher performance of bilinguals in letter fluency. Bilinguals are also suggested to perform better in phonemic fluency tasks when these require added executive processing, such as task-switching activity^40^.

The effects of bilingualism thus are moderated by the task on which monolinguals and bilinguals are compared. Particularly, a bilingual (or multilingual) advantage is more likely to be observed on nonverbal tasks that put increased demands on attentional capacities, such as interference suppression, conflict monitoring, and task-switching paradigms.

Findings contesting the differences between bi- and monolinguals in some of the cognitive domains listed above have been presented by two fairly large meta-analyses by Donnelly et al.^9^ and Lehtonen et al.^10^ (see also Goldsmith & Morton^46^, for a discussion). Both meta-analyses reported very small effects (Hedge’s *g* ≈ 0.10) of bilingualism on inhibitory control, concept shifting, and working memory. However, these effects mostly vanished when corrected for publication bias. An exception to this was the interference cost in inhibition control tasks, for which the meta-analysis by Donnelly et al.^9^ found higher robustness in terms of a bilingual advantage. Of note, however, the methodology of these meta-analyses was criticized in more recent meta-analyses^20,27^ for using a publication bias correction that may have led to an underestimation of the true effect size. The meta-analysis by Lehtonen et al. was additionally criticized for removing outlier studies with large effect sizes (favoring a bilingual advantage) without methodological justification and for including verbal processing tasks in the calculation of the overall effect size.

Some limitations of the research on the association of multi-language abilities with cognitive functions additionally exacerbate the comparability of findings. For one, and most frequently noted, there is the confounding of bi- and multilingualism with other variables affecting the cognitive reserve, such as educational attainment, socio-economic status, and immigration status^47–49^. Second, a central conclusion has been that the bilingual advantage in some cognitive tasks is essentially an effect that results from the interaction of person characteristics (e.g. age and language proficiency) and control demands of the task tested. Accordingly, superior performance of bilinguals is most often found in children and older adult samples for nonverbal tasks with increased executive or attentional control demands^7,15^. Third, the definition and operationalization of bilingualism vary between studies, which complicates the isolation of possible differential effects of the number and proficiency of languages spoken.

### The present study

The present study investigated the effects of multi-language abilities (i.e. bi- and multilingualism) on multiple cognitive functions in older age. In doing so, two shortcomings of earlier studies testing the bilingual advantage hypothesis are addressed: (a) controlling for confounding effects, and (b) restricting the investigation to a sample of adults over the age of 60 years, for whom positive effects of speaking multiple languages have been more consistently found. We used data from a large subsample of the 1000BRAINS^50^ study, a German cohort study that provides data on language skills and performance on various cognitive tests.

Based on the supportive evidence regarding the advantage of multi-language abilities, we assumed that older bi- and multilinguals—in this study referred to as the essentially multilingual group—would exceed older monolinguals in their cognitive performance. Specifically, in line with the bilingual advantage hypothesis, we expected the multilingual group to be superior to monolinguals in interference suppression, working memory, and concept shifting, but not necessarily verbal fluency. To investigate the effects of bi- and multilingualism independent of potentially confounding effects of participants’ educational attainment (which are confounded with multi-language abilities acquired in school or language training contexts) and restrictions in their daily instrumental and social activity, we included these variables as covariates. Furthermore, we explored whether an earlier age of second language acquisition and a higher level of language proficiency (across all languages spoken) is positively correlated with older multilinguals’ cognitive performance.

Unlike studies examining the effects of lifelong bilingualism—Which is usually operationalized as the simultaneous acquisition of both languages in early age^11^—The present study used a less strict operationalization of multi-language ability, according to which bi- and multilingual education within school or language training contexts were also considered. This way, more understanding can be gained about how language learning in a non-native language context, an increasingly prevalent phenomenon, affects cognition in older age.

## Results

### Comparisons of cognitive performance of bi- and multilinguals

Preliminary non-parametric Kruskal–Wallis tests (conducted because the assumption of normal distribution was violated for education and age) revealed that multilinguals were significantly more educated, χ^2^(1) = 96.06, *p* < 0.001, η^2^ = 0.181, and less restricted in their daily activity, χ^2^(1) = 15.06, *p* < 0.001, η^2^ = 0.027, than monolinguals. There was also a statistically significant but negligibly small difference in age between groups, χ^2^(1) = 5.15, *p* = 0.023, η^2^ = 0.008.

To investigate the effect of language group on performance in the five cognitive domains, while controlling for education level and restrictions in daily activity, we employed hierarchical Bayesian regression analyses (using the ‘brms’ package^51^). Our approach compared a restricted model featuring education level and restrictions in daily activity as sole predictors against an unrestricted model that further incorporated language group as a predictor.

**Figure 1:**
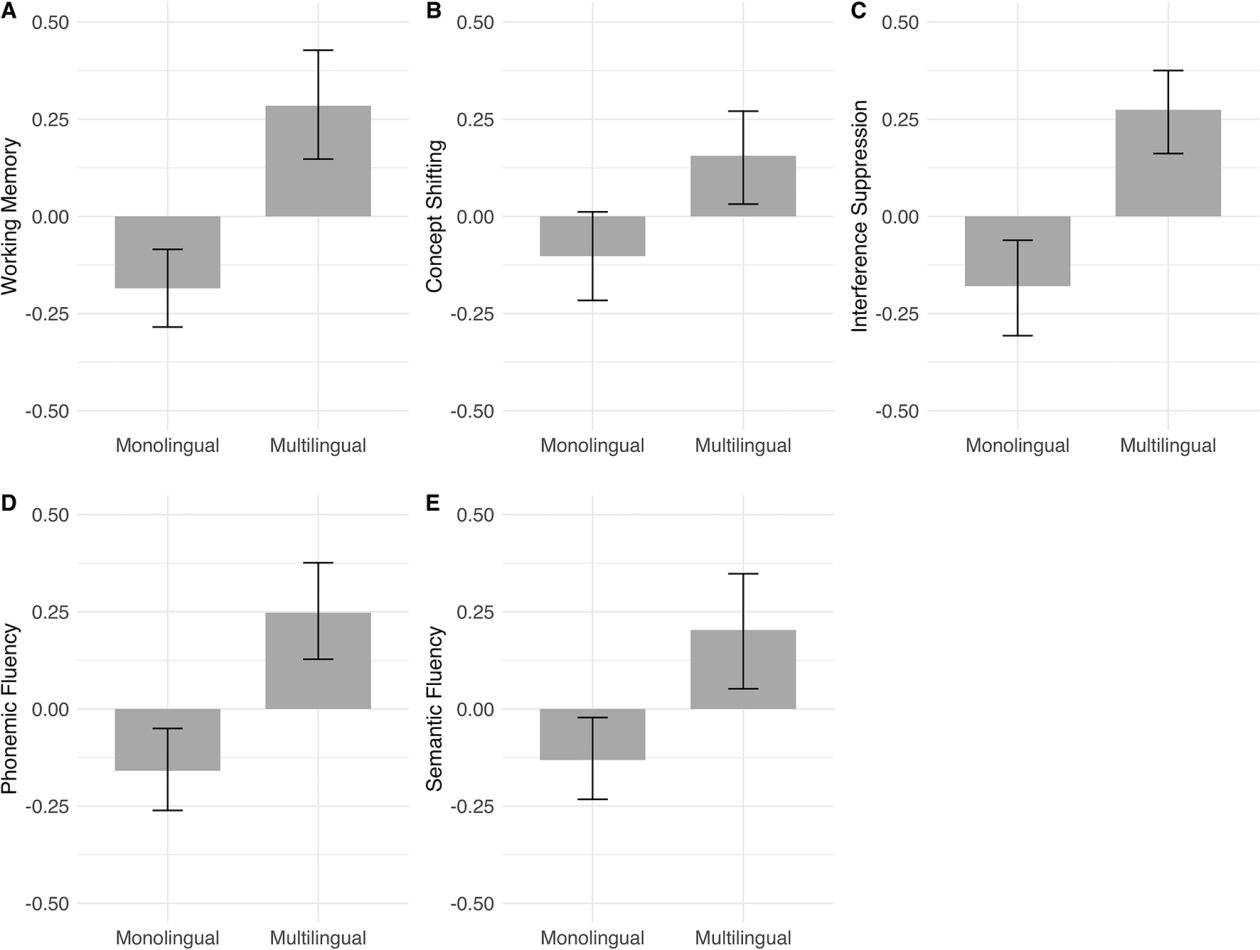
Mean differences (z-standardized) in cognitive performance between language groups in working memory (**A**), concept shifting (**B**), interference suppression (**C**), phonemic fluency (**D**), and semantic fluency (**E**)*.* Error bars indicate bootstrapped 95% confidence intervals. Only the group differences in working memory, interference suppression, and phonemic fluency remained meaningful after controlling for education level and restrictions in daily activity.

We applied an uninformative flat prior across the real numbers to estimate the population-level effects of education level, restrictions in daily activity, and language group. Figure 1 shows the mean differences between language groups in the five cognitive domains.

Small incremental effects of language group were found on working memory (∆*R*^2^ = 0.007), interference suppression (∆*R*^2^ = 0.018), and phonemic fluency (∆*R*^2^ = 0.008), but not on concept shifting or semantic fluency. Particularly, there was anecdotal evidence for a higher performance of multilinguals on working memory (BF10 = 1.69) and phonemic fluency (BF10 = 1.61), and there was strong evidence for a higher performance of multilinguals on interference suppression (BF10 = 27.75). Evidence for a null effect, that is, no incremental effect of multilingualism, was provided in the case of concept shifting (BF10 = 0.40) and semantic fluency (BF10 = 0.47). The effects of language group on each of the five cognitive domains, controlling for the effects of education level and restrictions in daily activity, are presented in Table 1.

**Table 1:** Results of the Bayesian regression analyses: model parameters of unrestricted models.

Outcome	*N*	Predictors (regression weights with 95% CIs in brackets)				*R*^2^ (∆*R*^2^)	BF_10_
		Intercept	Education level	Restrictions in daily activity	Language group		
Working memory	503	– 0.07 (– 0.18; 0.04)	0.30 (0.21; 0.39)	0.12 (0.04; 0.20)	**0.19** (0.00; 0.37)	0.152 **(0.007)**	**1.69**
Concept shifting	501	– 0.04 (– 0.15; 0.08)	0.21 (0.11; 0.31)	0.02 (– 0.07; 0.11)	0.10 (– 0.10; 0.29)	0.059 (0.003)	0.40
Interference suppression	501	– 0.13 (– 0.24; – 0.02)	0.14 (0.05; 0.24)	0.16 (0.07; 0.24)	**0.30** (0.11; 0.49)	0.100 **(0.018)**	**27.75**
Phonemic fluency	499	– 0.07 (– 0.18; 0.04)	0.21 (0.12; 0.31)	0.11 (0.03; 0.20)	**0.19** (0.00; 0.38)	0.099 **(0.008)**	**1.61**
Semantic fluency	502	– 0.05 (– 0.16; 0.07)	0.20 (0.10; 0.29)	0.19 (0.11; 0.28)	0.11 (– 0.08; 0.30)	0.109 (0.004)	0.47

Language group coefficients indicate the difference in conditional group means (Monolingual = 1, Multilingual = 2), with monolinguals as the reference group. Values in bold indicate effects of language group for which the data-based evidence points toward meaningful contributions of the variable to cognitive performance. 95% credible intervals show the intervals of highest density in the posterior distributions and indicate the precision of the estimates.

∆*R*^2^ = Increment in *R*^2^ due to the addition of language group into the model. BF_10_: Bayes factor indicating the data-based evidence for the hypothesis that speaking multiple languages exhibits a cognitive effect beyond participants’ education and daily activity levels; 1 ≤ BF_10_ < 3: anecdotal evidence, 3 ≤ BF_10_ < 10: moderate evidence, 10 ≤ BF_10_ < 30: strong evidence^87^; BF_10_ < 1 provides evidence for the null hypothesis that language group shows no effect beyond participants’ education and daily activity levels.

As the working memory factor included six tasks (two spatial, two digit span, and two visual memory tests), which likely vary in their attentional demands and complexity, we conducted additional regression analyses on these six working memory tasks. The results are presented in Table A1 of the online supplement. They show small advantages of multilinguals on the backward digit span task (∆*R*^2^ = 0.020, BF10 = 39.28) with very strong evidence, and the Benton visual memory task (∆*R*^2^ = 0.009, BF10 = 2.85) with anecdotal evidence. There was hardly any to no evidence of a multilingual advantage in the remaining working memory tasks.

### Associations of age of acquisition (AoA) of the second language and language proficiency with cognitive performance of multilinguals

To explore whether an earlier AoA of the second language and a higher level of language proficiency across all languages spoken were associated with better cognitive performance among bi- and multilinguals (*N* = 208), correlations were computed for each cognitive domain. The mean AoA was *M* = 14.97 (*SD* = 10.34), with 4% of the bi- and multilingual participants reporting that they had started to acquire their second language before the age of 6, 43% between ages 6 and 11, 31% between ages 12 and 19, and 17% after age 20 (4% did not provide data on this). Furthermore, the mean language proficiency level across all languages spoken was *M* = 3.40 (*SD* = 0.48, range: 3 to 5).

In the online supplement, Appendix B provides the correlations of performance in the cognitive domains with AoA (Table B1) and with the mean language proficiency level (Table B2), with bootstrapped confidence intervals. As can be taken from Table B1, no statistically significant correlations were found between AoA and any of the five cognitive domains, as indicated by the bias-corrected bootstrapped 95% confidence intervals which all contain a null correlation. Similarly, no statistically significant correlations emerged between the mean language proficiency and performance in any of the five cognitive domains (see Table B2).

## Discussion

To contribute to a better understanding of whether the bilingual—and here by extension multilingual—advantage hypothesis can be supported or not, the current study compared the performance of German monolinguals and multilinguals older than 60 years in several cognitive domains. Participants’ educational attainment and restrictions in daily (cognitively stimulating) activity, suspected to be confounded with multi-language mastery and cognitive performance, were controlled for by including these variables as covariates in the analyses.

Multilinguals outperformed their monolingual peers in working memory when educational attainment and restrictions in daily activity were held constant. The evidence concerning this effect on the composite factor of working memory was merely anecdotal in our study. However, the effect was substantively higher when looking at the performance in the backward digit span task alone. In line with the findings of Comishen and Bialystok^34^, this is well explained by the increased attentional demands required in recalling a sequence of numbers in reverse order, as compared to recalling it in forward or random order. As the activation and switching between languages supposedly train attentional resources, benefits of bi- and multilingualism emerge in cognitive tasks with high attentional demands^34^. The other subtests of the working memory domain (specifically, the block-tapping and Visual Patterns tasks) were probably less demanding in attentional resources, which might explain why the effect of the composite working memory factor was comparatively small. Importantly, this demonstrates that the effects of bi- and multilingualism can also differ within executive function domains such as working memory depending on the specific demands of the cognitive task and the cognitive processes targeted by the task^15^.

Moreover, multilinguals outperformed monolinguals with regard to interference suppression when controlling for the covariates, which replicates one of the most consistent findings in the literature. The finding of an incremental positive effect of the multilingual group on phonemic fluency (only supported by anecdotal evidence in the present study), but no group effect on semantic fluency is likely explained by previous findings that phonemic fluency requires more executive control than semantic fluency^39,40,43^. Furthermore, we found no effect of language group on concept shifting in the TMT. This might be due to the measure being created as the difference between TMT task A, which measures visual tracking and motor speed, and TMT task B, which primarily measures concept shifting^52^. Thus, visual-motor tracking skills, for which language group differences are not indicated, and shifting ability were confounded in this variable. This also means that a conclusion about differences between the groups in concept shifting (alone) cannot be readily drawn based on these results.

Consistent with recent meta-analytic findings^9,20^ and much like other experience-related effects on executive function performance, the observed incremental effect of multi-language abilities on cognitive performance beyond education and daily activity levels was very small in magnitude (∆*R*^2^ < 0.03). That is, small enough to not be noticeable in everyday cognitive tasks. However, more importantly, these small effects have been shown to translate to practically relevant differences in neuroplasticity resulting from the bi- or multilingual experience^27^, for instance, in terms of delaying the onset of symptoms of dementia^26^. Similar to physical as well as cognitive training programs, language learning programs, thus, might provide a valuable supplementary option for building cognitive reserve^53^, because language learning involves an extensive brain network otherwise subject to age-related cognitive decline. Our results suggest a task- or function-specific rather than a function-general impact of multilingualism on the cognitive performance of older adults. This conclusion is in line with findings of previous studies (comparing monolinguals to bilinguals exclusively), showing that the cognitive benefits of multi-language abilities do not generalize across aspects of executive functioning^19,20^.

The correlative analyses yielded no substantive support for the assumption that an earlier acquisition of the second language and a higher mean language proficiency across the languages spoken in the multilingual group are associated with better cognitive performance. The lack of statistically significant findings here is at odds with some studies that reported moderating effects of these variables on the performance in executive function tasks^54,55^. However, it is in line with other studies^10,56^ that also reported no moderating effects of second language proficiency or AoA on performance in executive function tasks in samples of older adults. There are various possible explanations for this lack of effect. Regarding language proficiency, we observed a rather small variance in this variable in the group of multilinguals, which likely restricted correlations with other variables. The effects of language proficiency might, thus, look different in a sample with higher heterogeneity on this measure. Other studies have pointed out the importance of the frequent and balanced usage of and exposure to the languages known^55–57^ to exercise the control of multiple languages and reap its benefits. Unlike children and younger adults, who spend a lot of time in bi- or multilingual environments (through school, work, family, etc.), older multilinguals are more likely to spend time in essentially monolingual environments with fewer opportunities to practice their other languages once they have settled down. Then neither an early AoA nor high language proficiency should be very helpful in further boosting cognitive performance in executive function tasks.

Among other moderating variables that are considered to influence the effects of multi-language ability, the typological difference between the languages spoken has been discussed. Antoniou and Wright^53^, for example, suggested two contrasting roles that language typology might play in the development of cognitive reserve resulting from foreign language learning: For one, the acquisition of languages with low linguistic similarities (e.g. English and Chinese) might require more cognitive effort^58^, which could result in more cognitive advantages later on. In contrast, languages with high linguistic similarities (e.g. Spanish and Italian) could be more rapidly learned, which, once a certain level of language proficiency is achieved, might lead to greater competition between these languages relative to dissimilar languages. This linguistic similarity would in turn require greater inhibitory effort and place greater demands on executive functions.

Lastly, we want to address some limitations concerning the present study. First, a bidirectional nature of the effect between multilingualism and cognitive functions can be assumed. That is, knowing multiple languages might influence individual levels of cognitive control, but the reverse—an enhancement of language abilities caused by high baseline cognitive ability—might also be true^59^. The correlative nature of our analyses does not allow for insights into the causal direction of the observed effects the way genuine experiments (with people randomly assigned to language learning courses and control conditions) or longitudinal studies would. However, drawing on previous longitudinal evidence^39,60^, it can be assumed that multi-language abilities predict cognitive function in older age to a substantive degree.

Second, measures of language proficiency based on self-reports are prone to inaccurate assessments due to their subjective nature^48^. Aside from standardized tests such as lexical decision tasks or verbal fluency tasks, such subjective measures are often used due to their cost-effectiveness. The LEAP-Q^61^, which was used in the 1000BRAINS study, is a comparably comprehensive self-report-based language assessment tool; the validity of the self-report measures has been mainly established by their high correlations with standardized tests of linguistic performance. However, Gullifer et al.^62^ point out the importance of additionally including more objective assessments of language performance when assessing the verbal abilities of bilinguals and multilinguals, such as verbal fluency tests in all languages spoken (In 1000BRAINS, these are only collected for the German language).

Third, in this study, classification into language groups was based on a very inclusive criterion, resulting in still highly heterogeneous groups in terms of individual language experiences. Consequently, no distinction was made between those who learned additional languages in schooling or language training contexts and those who learned these languages in a native-speaking context. Both constitute very different language experiences with implications for language usage that have differential cognitive impacts^27^. This study’s findings, which show some impact of multi-language abilities despite these experiential differences, highlight the multifaceted nature of the effect and, importantly, validate the benefits of language acquisition outside of the native language learning context. Furthermore, the definition and operationalization of bilingualism varies across studies. It is sometimes restricted to the knowledge of exactly two languages, while other times it is extended to the knowledge of more than two languages, or a continuous conceptualization^63–65^ is used that places individuals on a “spectrum of knowledge of two (or more) languages”^66^. The latter goes along with more recent attempts of conceptualizing bilingualism/multilingualism as a multidimensional construct of various experiences (e.g. in terms of the degree of exposure to the languages and the dominance and balance of language use) that influence language use and proficiency^62,67^. Moving forward, such a multidimensional approach will serve to portray multilingualism more realistically in its complexity.

## Method

### Participants

Data from the 1000BRAINS study (1000BRAINS; Caspers et al.^50^) were used for the present analyses. 1000BRAINS is an ongoing German longitudinal cohort study of the Jülich Research Centre in cooperation with the Heinz Nixdorf Recall Study (HNR) and the HNR MultiGeneration Study^68,69^. Among various other neuropsychological and physiological data, 1000BRAINS records data on language skills and tests of cognitive-executive functions.

For the current study, we used demographic, cognitive, and language-related data from the cohort aged 60 years and older (*N* = 787 participants). In the current sample, subjects were included when they had completed the Language Experience and Proficiency Questionnaire (LEAP-Q; Marian et al.^61^), reported having had no difficulties in their language development (e.g. dyslexia), and had completed cognitive tests in the domains of working memory, attention, verbal fluency, concept shifting, cognitive flexibility, and interference suppression. Based on these criteria, 249 participants were excluded (of which 208 had not completed the LEAP-Q). Ten participants suspected to exhibit mild cognitive impairment, as reflected in values equal to or lower than 8 on the DemTect scale^70^, were further excluded from the analyses.

The final sample consisted of *N* = 528 participants (44% female) with a mean age of *M* = 68.97 years (*SD* = 5.78; range: 60 to 85 years). Based on the LEAP-Q, 320 (60.6%; thereof 48% female) participants of the sample were classified as monolingual (i.e. German-speaking only) and 208 participants (39.4%; thereof 39% female) were classified as essentially multilingual, that is, speaking at least two languages. Among the multilinguals, 94% reported German as their mother tongue. In the multilingual group, 136 participants reported speaking two languages, 58 reported speaking three languages and 14 reported speaking four languages. Furthermore, 74% of the multilinguals reported having started acquiring their second language between the ages of 6 to 19 years. Table 2 presents participants’ age, education level, level of restrictions in daily activity, and performances on the cognitive domains stratified by language group.

**Table 2:** Age, education, restrictions in daily activity, and cognitive performance (z-standardized) of mono- and multilinguals.

Variable	Monolinguals (*n* = 320)		Multilinguals (*n* = 208)	
	*M*	*SD*	*M*	*SD*
Age	69.48	6.00	68.19	5.34
Education level	5.88	1.78	7.59	1.91
Restrictions in daily activity	54.55	3.09	55.60	2.30
Working memory	– 0.19	0.94	0.29	1.03
Concept shifting	– 0.10	1.02	0.16	0.95
Stroop	– 0.18	1.08	0.27	0.79
Phonemic fluency	– 0.16	1.00	0.25	0.95
Semantic fluency	– 0.13	0.97	0.20	1.01

*N* = 528. Sample range of education level: 3 to 10 (Higher scores indicate higher educational attainment). Sample range of levels of restriction in daily activity: 36 to 60 (Higher scores indicate fewer restrictions in daily activity). The groups differed significantly in education, χ^2^(1) = 96.06, *p* < 0.001, η^2^ = 0.181, and their restrictions in daily activity, χ^2^(1) = 15.06, *p* < 0.001, η^2^ = 0.027.

### Measures

#### Language proficiency

The *Language Experience and Proficiency Questionnaire* (LEAP-Q, Marian et al.^61^; German translation by Weigel & Gonzalez-Marquez^71^) assesses language profiles of healthy mono-, bi-, and multilinguals speaking up to five languages (including up to two mother tongues in the 1000BRAINS study). It is a self-report questionnaire assessing the level of linguistic proficiency and age of language acquisition with respect to the domains of speaking, comprehension, reading, and writing, as well as the contexts and degrees of language usage. The level of language proficiency in speaking, comprehension, reading, and writing is assessed for each language on a 5-point scale based on self-report (1 = none, 2 = low, 3 = adequate, 4 = good, 5 = very good). For the classification into language groups, participants who reported at least adequate proficiency in speaking and comprehension of two or more languages were classified as essentially multilingual, and participants who reported no or low proficiency in any other language than German were classified as monolingual.

#### Cognitive measures

Data from 12 cognitive tests (including subtests of the same inventories, all administered in German) were considered (for a complete list, see Table A3 in the online supplement). To aggregate the working memory and the verbal fluency measures into superordinate factors (allowing for more efficient use of test power in the subsequent analyses), we used a factor-analytic approach. Values of cognitive tests measuring reaction times were reverse-coded for better interpretability. The data analyses were conducted with R (R Core Team^72^). For confirmatory factor analyses, the R package ‘lavaan’^73^ was used.

**Figure 2:**
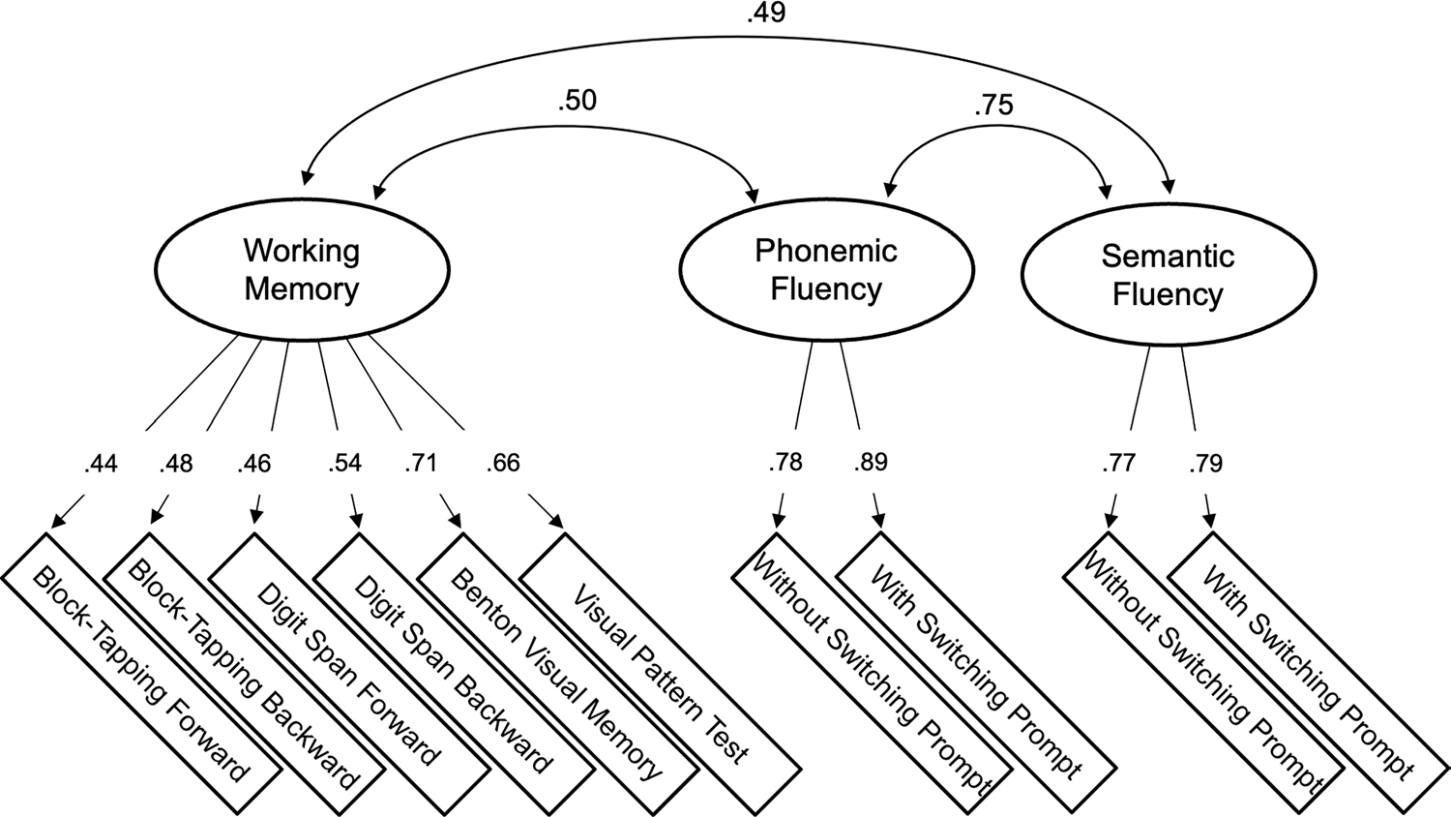
Schematic Representation of the Confirmatory Factor Model. Single arrows show the factor loadings and curved double arrows show the latent factor correlations. Model fit indices: χ^2^ (*df* = 32, *N* = 528) = 104.60, *p* < 0.001, CFI = 0.949, RMSEA = 0.066, SRMR = 0.044.

A parallel analysis^74^ suggested a three-factor solution for the 10 working memory and verbal fluency measures. In a maximum likelihood factor analysis with *oblimin* rotation, these three factors explained 46% of the variance in the variables. In a subsequent confirmatory factor analysis using a robust maximum likelihood estimator, theoretically sensible modifications were made so that all measures of working memory loaded on one factor, and the two measures of phonemic fluency and the two measures of semantic fluency loaded on one factor, respectively. The underlying factor model presented a good fit to the data (by recommendations of Hu & Bentler^75^): χ^2^ (*df* = 32, *N* = 528) = 104.60, *p* < 0.001, CFI = 0.949, RMSEA = 0.066, SRMR = 0.044. Please see Fig. 2 for the factor solution (i.e. factor loadings and factor correlations). The internal consistencies were McDonald’s ω = 0.72 for working memory, ω = 0.82 for phonemic fluency, and ω = 0.75 for semantic fluency. A Stroop task and a concept-shifting task were additionally included as individual indicators of interference suppression and cognitive flexibility, respectively. In the online supplement, Table A2 provides the zero-order correlations of the 12 cognitive subtests, language group, education level, and restrictions in daily activity.

##### Working memory

This domain comprised six subtests. Two measures of spatial memory span (forward and backward) based on the “Corsi Block-Tapping-Test” (CBT; Schellig^76^) were used. Reliability estimates for the immediate block span in this task were reported to range from *r* = 0.94 to *r* = 0.97^77^. The “Visual Patterns Test” (VPT; Jülich version, adapted from Della Sala et al.^78^) measures short-term visual memory. Participants are asked to reproduce (increasingly complex) matrix patterns on a blank grid. Visual memory was assessed with the “Benton-Test” (Benton Sivan et al.^79^), in which participants are asked to correctly memorize and reproduce geometric patterns of 20 items (Cronbach’s α = 0.90^79^). To assess numerical memory, the forward and backward digit span tests from the “Nuremberg Geronto-Psychological Inventory” (NAI; Oswald & Fleischmann^80^) were applied.

##### Phonemic and semantic fluency

These domains each comprised two subtests of the “Regensburg Verbal Fluency Test” (Aschenbrenner et al.^81^) assessing both formal lexical word fluency (phonemic fluency) and semantic word fluency (semantic fluency). For both, one measure without and one with a switching prompt is included. In the basic phonemic fluency task, the participant is asked to produce as many words as possible beginning with a given initial letter within two minutes, while in the phonemic fluency “switching” task the participant is asked to produce words alternating between two different initial letters within two minutes. For the respective semantic fluency task, the phonemic tasks are repeated with a focus on one (basic task) or two (switching task) given categories.

##### Concept shifting

Concept shifting ability was assessed by the difference in time spent for tasks B and A of the “Trail Making Test” (TMT; Morris et al.^82^). In the TMT, randomly arranged numbers (TMT task A) or numbers and letters (TMT task B) have to be connected in their consecutive order (alternating between numbers and letters in task B). Satisfying reliabilities between *r* = 0.78 and *r* = 0.92 have been reported for the TMT^83^.

##### Interference suppression

Interference suppression was assessed by the Jülich version of an interference inhibition test similar to the Stroop task^84^, adapted from Bäumler^85^, measuring susceptibility to interference (in reaction time) in nominating a color word and nominating the color that the word is printed in, without reading the color word itself.

#### Covariates

We used educational attainment and restrictions in daily activity as covariates for our analyses. Educational attainment was assessed with the “International Standard Classification of Education” (ISCED; UNESCO Institute for Statistics^86^) on eight broad levels, from early childhood/primary education to the doctoral or an equivalent level. Since these education levels are roughly reflected in the number of years of education, we treated the variable as continuous in our analyses. As a measure of the daily activity level, the “Nuremberg Geriatric Activity Scale” (Nürnberger-Alters-Alltagsskala-Skala) from the Nuremberg Geronto-Psychological Inventory^80^ was used. Using 20 self-report items, it assesses individual restrictions in areas of daily instrumental activities, social activities, and memory performance (e.g. while shopping, watching TV, or cleaning up). Higher sum scores (on a score range of 20 to 60 points) reflect fewer restrictions and a higher activity level in everyday activities.

#### Age of acquisition (AoA) of the second language and language proficiency

As a measure of second language AoA, we calculated a mean AoA score in speaking and comprehension of the secondary language with the highest proficiency. For both bi- and multilinguals, this was the language listed in second place in the LEAP-Q^61^.

As a measure of the level of language proficiency, mean proficiency levels in speaking and comprehension were calculated for the second mother tongue, first foreign language, second foreign language, and third foreign language. These proficiency levels were subsequently aggregated across the reported languages, resulting in an overall mean language proficiency score.

### Ethics declaration concerning human subjects

In this study, data from the German 1000BRAINS study were reanalyzed. Experiments and data collection for the 1000BRAINS study were performed in accordance with the Declaration of Helsinki and research methods followed relevant guidelines/regulations and standard procedures implemented in ongoing brain imaging studies. Informed consent was obtained from all subjects and/or their legal guardians. The 1000BRAINS study protocol was approved by the ethics committee of the University of Duisburg-Essen (Germany).

## Supplementary Information

Supplementary Tables.

## Data Availability

The raw data supporting the findings of this study were used under a license granted by the Jülich Research Centre. The data are part of a large ongoing research project and are not made publicly available at this stage. However, we provide the correlation matrix and analysis syntax to replicate the results of the factor analysis via an OSF repository at https://osf.io/uq874/. R-syntax of the descriptive and regression analyses can be additionally requested from P.A.-A.
