# Supplementary material for: Multilingualism is associated with small task-specific advantages in cognitive performance of older adults
Source: Sci Rep. 2023 Oct 7;13:16912. doi: 10.1038/s41598-023-43961-7 (PMC10560281; doi:10.1038/s41598-023-43961-7)
Supplement: Supplementary file 1 — Supplementary Tables. [file 41598_2023_43961_MOESM1_ESM.docx]

**Multilingualism is associated with small task-specific advantages in cognitive performance of older adults**

**Online Supplement**

Priscilla Achaa-Amankwaa, Ekaterina Münch, Hanna Miksch, Johanna Stumme, Stefan Heim, and Mirjam Ebersbach

**Appendix A**

**Regression results for working memory tasks, zero-order correlations and list of neuropsychological performance tests**

| **Outcome** |  | **Predictors (regression weights with 95% CIs in brackets)** | | | |  | ***R*^2^ (∆*R*^2^)** | **BF_10_** |
| --- | --- | --- | --- | --- | --- | --- | --- | --- |
|  | *N* | Intercept | Education level | Restrictions in daily activity | Language group |  |  |  |
| Block-Tapping Forward | 503 | 0.01  (-0.11; 0.12) | 0.06  (-0.03; 0.16) | 0.04  (-0.05; 0.13) | -0.01  (-0.21; 0.18) |  | .010 (.002) | 0.26 |
| Block-Tapping Backward | 503 | 0.03  (-0.09; 0.15) | 0.2  (0.11; 0.30) | 0.06  (-0.03; 0.15) | -0.05  (-0.25; 0.14) |  | .048 (.002) | 0.29 |
| Digit Span Forward | 503 | -0.06  (-0.17; 0.06) | 0.14  (0.05; 0.24) | 0.09  (-0.00; 0.17) | 0.17  (-0.03; 0.36) |  | .053 (.007) | 1.05 |
| Digit Span Backward | 503 | -0.12  (-0.23; -0.01) | 0.15  (0.06; 0.24) | 0.07  (-0.02; 0.15) | **0.31**  (0.12; 0.50) |  | .081 **(.020)** | **39.28** |
| Benton Visual Memory | 492 | -0.09  (-0.20; 0.02) | 0.31  (0.22; 0.40) | 0.16  (0.07; 0.24) | **0.21**  (0.03; 0.39) |  | .185 **(.009)** | **2.85** |
| Visual Pattern Test | 502 | -0.03  (-0.14; 0.09) | 0.29  (0.20; 0.38) | 0.05  (-0.04; 0.13) | 0.09  (-0.10; 0.28) |  | .106 (.003) | 0.39 |
| **Table A1**. Results of Bayesian regression analyses for working memory tasks: Model parameters of unrestricted models. Language group coefficients indicate the difference in conditional group means (Monolingual = 1, Multilingual = 2), with monolinguals as the reference group. Values in bold indicate effects of language group for which the data-based evidence points toward meaningful contributions of the variable to cognitive performance. 95% credible intervals show the intervals of highest density in the posterior distributions and indicate the precision of the estimates.  ∆*R*^2^ = Increment in *R*^2^ due to the addition of language group into the model. BF_10_: Bayes factor indicating the data-based evidence for the hypothesis that speaking multiple languages exhibits a cognitive effect beyond participants’ education and daily activity levels; 1 ≤ BF_10_ < 3: anecdotal evidence, 3 ≤ BF_10_ < 10: moderate evidence, 10 ≤ BF_10_ < 30: strong evidence, 30 ≤ BF_10_ < 100: very strong evidence^80^; BF_10_ < 1 provides evidence for the null hypothesis that language group shows no effect beyond participants’ education and daily activity levels. | | | | | | | | |

| Variable | | 1 | 2 | 3 | 4 | 5 | 6 | 7 | 8 | 9 | 10 | 11 | 12 | 13 | 14 | 15 |
| --- | --- | --- | --- | --- | --- | --- | --- | --- | --- | --- | --- | --- | --- | --- | --- | --- |
| 1 | Language Group |  | .040 | .044 | .044 | .044 | .043 | .043 | .043 | .043 | .043 | .043 | .043 | .043 | .043 | .043 |
| 2 | Education Level | .42 |  | .044 | .043 | .043 | .043 | .043 | .041 | .041 | .043 | .043 | .043 | .042 | .042 | .043 |
| 3 | Restrictions Daily Activity | .18 | .17 |  | .045 | .044 | .044 | .044 | .044 | .044 | .045 | .044 | .044 | .044 | .044 | .044 |
| 4 | Block-Tapping Forward | .04 | .09 | .05 |  | .042 | .043 | .042 | .043 | .041 | .043 | .043 | .044 | .044 | .043 | .043 |
| 5 | Block-Tapping Backward | .05 | .19 | .09 | .30 |  | .043 | .042 | .042 | .041 | .043 | .043 | .043 | .043 | .043 | .043 |
| 6 | Digit Span Forward | .16 | .19 | .12 | .15 | .13 |  | .040 | .042 | .042 | .043 | .042 | .042 | .042 | .043 | .043 |
| 7 | Digit Span Backward | .21 | .22 | .12 | .25 | .24 | .39 |  | .041 | .042 | .042 | .042 | .043 | .042 | .043 | .043 |
| 8 | Benton Visual Memory | .24 | .37 | .23 | .26 | .33 | .30 | .35 |  | .038 | .040 | .042 | .042 | .042 | .042 | .042 |
| 9 | Visual Pattern Test | .18 | .31 | .11 | .36 | .36 | .27 | .30 | .49 |  | .042 | .043 | .043 | .043 | .043 | .043 |
| 10 | Concept Shifting | .13 | .21 | .06 | .17 | .20 | .22 | .26 | .42 | .29 |  | .042 | .042 | .042 | .042 | .043 |
| 11 | Interference Suppression | .22 | .23 | .20 | .11 | .13 | .23 | .26 | .32 | .23 | .30 |  | .042 | .042 | .042 | .042 |
| 12 | Phonemic Fluency | .19 | .21 | .14 | .08 | .16 | .29 | .24 | .31 | .17 | .25 | .29 |  | .032 | .039 | .039 |
| 13 | Phonemic Fluency Switch | .18 | .27 | .16 | .12 | .21 | .30 | .31 | .33 | .21 | .30 | .30 | .69 |  | .038 | .037 |
| 14 | Semantic Fluency | .18 | .25 | .20 | .13 | .15 | .14 | .17 | .32 | .22 | .24 | .25 | .47 | .50 |  | .035 |
| 15 | Semantic Fluency Switch | .11 | .19 | .22 | .16 | .21 | .16 | .22 | .31 | .22 | .23 | .30 | .44 | .52 | .60 |  |
| **Table A2**. Zero-order correlations of independent and dependent variables on the lower triangle and standard errors on the upper triangle. *N* = 528. Language group: monolingual = 1, multilingual = 2. All correlations >.12 are significant at the Holm-adjusted *p*-value*.* | | | | | | | | | | | | | | | | |

| **Test** | **Functions** | **Description** |
| --- | --- | --- |
| Trail Making Test A and B (taken from CERAD-Plus) (Morris et al., 1989) | A: Visual attention, processing speed  B: Concept shifting (cognitive flexibility) | A: Connecting randomly arranged digits in ascending order  B: Alternately connecting numbers and letters in ascending order |
| Benton-Test (Benton et al., 2009) | Visual memory | Free recall of 20 figures |
| Block-Tapping-Test (Schellig, 1997) | Visuo-spatial working memory (forward, backward) | Repeating a by trial increasing sequence of blocks on a board of 9 blocks, in equal and reverse order |
| Visual Pattern Test (Jülich version) (similar to: Della Sala et al., 1997) | Visual working memory | Memorizing a matrix pattern of black and white squares in grids of increasing complexity |
| Digit Span Test (from Nürnberger Alters-Inventar) (Oswald and Fleischmann, 1997) | Verbal working memory (forward, backward) | Repeating a by trial increasing sequence of spoken numbers in equal and reverse order |
| Regensburg Verbal Fluency Test (RWT; Aschenbrenner et al., 2000) | Semantic /Phonemic verbal fluency | Producing words beginning with a given letter or words from specific categories |
| Color-Word Interference Test (Jülich version) (similar to Stroop, 1935; Bäumler, 1985) | Visual attention  Information processing speed  Susceptibility to interference | Reading words with color meaning; naming the color of colored boxes; naming the color of words with color meaning printed in a different color |
| **Table A3**. Neuropsychological performance tests included in this study’s analyses (as administered in 1000BRAINS). Test functions and descriptions adapted from Table 2 in Caspers et al. (2014, p.7). CERAD: Consortium to Establish a Registry for Alzheimer’s Disease. | | |

**Appendix B**

**Correlation analyses results**

| Cognitive Domain | *r* | bootstrapped 95%-CI | |
| --- | --- | --- | --- |
|  |  | lower | upper |
| Working Memory | -.094 | -.217 | .024 |
| Concept Shifting | -.049 | -.181 | .109 |
| Interference Suppression | -.001 | -.105 | .108 |
| Phonemic Fluency | -.011 | -.127 | .102 |
| Semantic Fluency | .017 | -.117 | .134 |
| **Table B1**. Correlations of cognitive performance with mean age of second language acquisition. *95%-*CI was calculated with bias-corrected nonparametric bootstrapping. *N*= 208. | | | |

| Cognitive Domain | *r* | bootstrapped 95%-CI | |
| --- | --- | --- | --- |
|  |  | lower | upper |
| Working Memory | -.053 | -.199 | .106 |
| Concept Shifting | -.028 | -.247 | .122 |
| Interference Suppression | .095 | -.029 | .210 |
| Phonemic Fluency | -.011 | -.173 | .139 |
| Semantic Fluency | -.119 | -.258 | .018 |
| **Table B2**. Correlations of cognitive performance with mean language proficiency across all languages spoken. *95%-*CI was calculated with bias-corrected nonparametric bootstrapping. *N* = 208. | | | |
